# Supplementary material for: Investigating the Role of Micromammals in the Ecology of Coxiella burnetii in Spain
Source: Animals (Basel). 2021 Mar 2;11(3):654. doi: 10.3390/ani11030654 (PMC8000606; doi:10.3390/ani11030654)
Supplement: Supplementary file 1 [file animals-11-00654-s001.pdf]

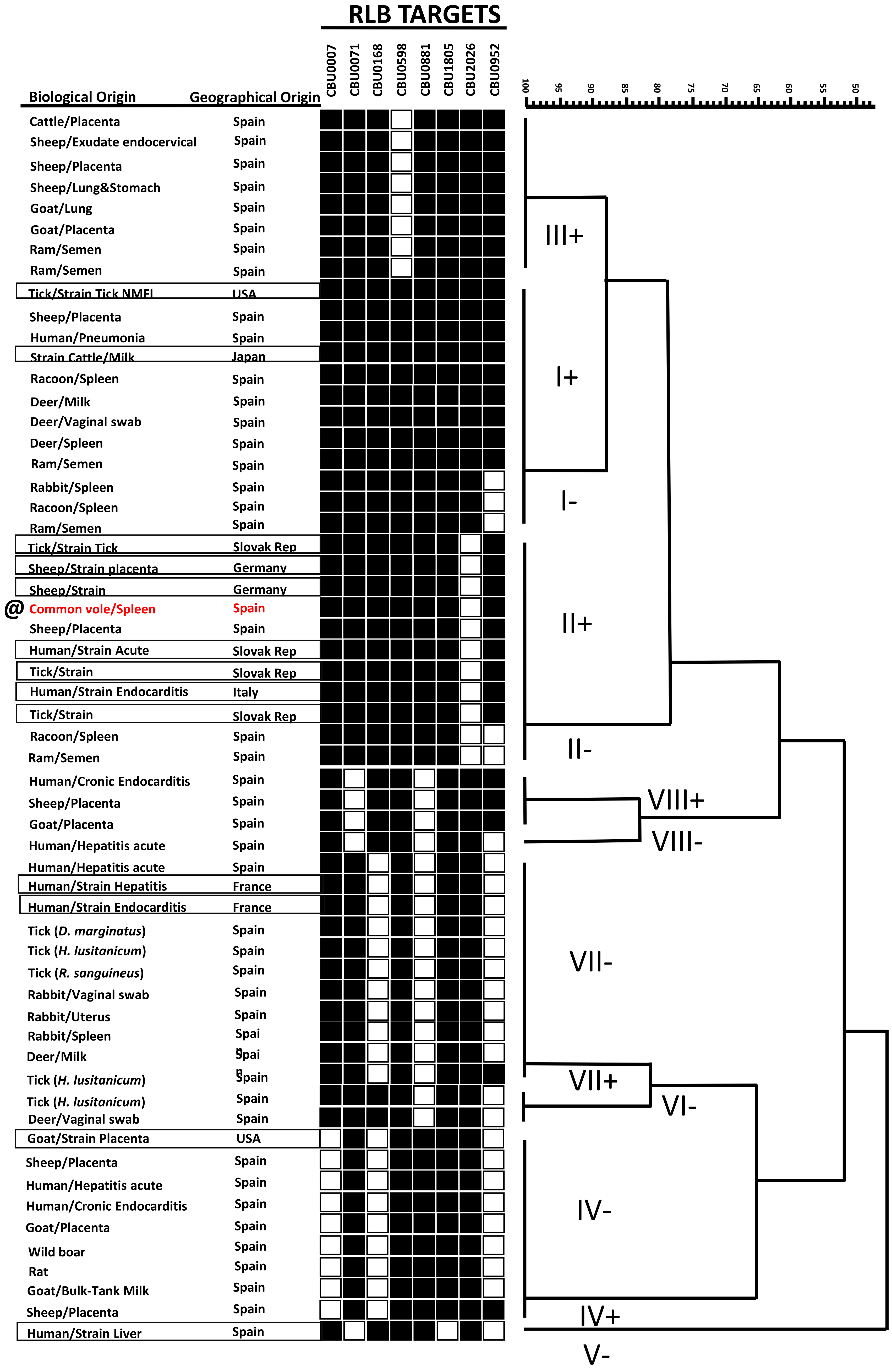

RLB TARGETS
CBU0007
CBU0071
CBU0168
CBU0598
CBU0881
CBU1805
CBU2026
CBU0952
Biological Origin
Geographical Origin
100
95
90
85
80
75
70
65
60
55
50
Cattle/Placenta Spain
Sheep/Exudate endocervical Spain
Sheep/Placenta Spain
Sheep/Lung&Stomach Spain
Goat/Lung Spain
Goat/Placenta Spain
Ram/Semen Spain
Ram/Semen Spain
Tick/Strain Tick NMFI USA
Sheep/Placenta Spain
Human/Pneumonia Spain
Strain Cattle/Milk Japan
Racoon/Spleen Spain
Deer/Milk Spain
Deer/Vaginal swab Spain
Deer/Spleen Spain
Ram/Semen Spain
Rabbit/Spleen Spain
Racoon/Spleen Spain
Ram/Semen Spain
Tick/Strain Tick Slovak Rep
Sheep/Strain placenta Germany
Sheep/Strain Germany
@ Common vole/Spleen Spain
Sheep/Placenta Spain
Human/Strain Acute Slovak Rep
Tick/Strain Slovak Rep
Human/Strain Endocarditis Italy
Tick/Strain Slovak Rep
Racoon/Spleen Spain
Ram/Semen Spain
Human/Cronic Endocarditis Spain
Sheep/Placenta Spain
Goat/Placenta Spain
Human/Hepatitis acute Spain
Human/Hepatitis acute Spain
Human/Strain Hepatitis France
Human/Strain Endocarditis France
Tick (D. marginatus) Spain
Tick (H. lusitanicum) Spain
Tick (R. sanguineus) Spain
Rabbit/Vaginal swab Spain
Rabbit/Uterus Spain
Rabbit/Spleen Spai
Deer/Milk Spai
Tick (H. lusitanicum) Spain
Tick (H. lusitanicum) Spain
Deer/Vaginal swab Spain
Goat/Strain Placenta USA
Sheep/Placenta Spain
Human/Hepatitis acute Spain
Human/Cronic Endocarditis Spain
Goat/Placenta Spain
Wild boar Spain
Rat Spain
Goat/Bulk-Tank Milk Spain
Sheep/Placenta Spain
Human/Strain Liver Spain
III+
I+
I-
II+
II-
VIII+
VIII-
VII-
VII+
VI-
IV-
IV+
V-
